# Supplementary material for: Dissolved organic matter thiol concentrations determine methylmercury bioavailability across the terrestrial-marine aquatic continuum
Source: Nat Commun. 2023 Oct 23;14:6728. doi: 10.1038/s41467-023-42463-4 (PMC10593767; doi:10.1038/s41467-023-42463-4)
Supplement: Supplementary file 1 — Supplementary Information [file 41467_2023_42463_MOESM1_ESM.pdf]

Supporting Information

Dissolved organic matter thiol concentrations determine methylmercury bioavailability across the terrestrial-marine aquatic continuum

Emily Seelen[1,2], Van Liem-Nguyen[3], Urban Wünsch[4], Zofia Baumann[1], Robert Mason[1], Ulf Skyllberg[5], Erik Björn[3]

1. University of Connecticut, Department of Marine Sciences, Groton, CT, USA
2. University of Southern California, Earth Sciences, Los Angeles, CA, USA (current location)
3. Umeå University, Chemistry Department, Umeå, Sweden
4. Technical University of Denmark, National Institute of Aquatic Resources, Section for Oceans and Arctic, 2800 Lyngby, Denmark
5. Swedish University of Agricultural Sciences, Department of Forest Ecology and Management Umeå, Sweden

Corresponding Author: Emily Seelen (e-mail: seelen@usc.edu), Erik Björn (e-mail: erik.bjorn@umu.se)

Table S1. Concentrations of dissolved organic sulfur, total Hg and MeHg at the different sampling sites.

State	Estuarine System	System Type	Latitude	Longitude	Temp. (C)	DOS (µM)	diss. HgT (pM)	diss. MeHg (pM)	%MeHg
DE	Delaware Bay	Marsh	39.578	-75.477	27.8	3.80	1.80	0.046	2.55
NJ	Berry Creek	Marsh	40.829	-74.080	24	8.59	45.45	2.78	6.11
CT	Pawcatuck River	Marsh	41.337	-71.876	30	7.95	8.00	0.44	5.49
MA	Merrimac River	Marsh	42.752	-70.837	23.4	7.87	6.17	0.45	7.28
CT	Housatonic River	River	41.315	-73.086	27.5	1.75	1.66	0.11	6.72
RI	Pawcatuck River	River	41.375	-71.832	25	1.83	NA	0.89	NA
CT	Connecticut River	River	41.383	-72.352	25.1	1.91	1.28	0.055	4.26
ME	Penobscot	River	44.690	-68.818	26.71	1.67	8.61	0.24	2.74
DE	Delaware Bay	Estuary	38.945	-74.971	22	2.05	1.29	0.001	0.08
NJ	Newark Bay	Estuary	40.670	-74.127	25.61	4.53	5.27	0.092	1.75
NY	Long Island Sound	Estuary	40.849	-73.782	25.3	2.40	1.09	0.034	3.11
CT	Housatonic River	Estuary	41.171	-73.114	26.7	2.34	0.98	0.027	2.80
CT	Connecticut River	Estuary	41.287	-72.351	23.3	1.53	1.81	0.079	4.36
MA	Buzzards Bay	Estuary	41.650	-70.913	27.2	0.00	0.68	0.020	2.99
MA	Merrimac River	Estuary	42.816	-70.821	18.3	2.99	1.21	0.15	12.35
MA	Merrimac River	Estuary	42.818	-70.878	23.8	2.06	1.54	0.014	0.93
ME	Penobscot	Estuary	44.432	-68.942	21.11	1.47	3.25	0.025	0.78
ME	Penobscot	Estuary	44.591	-68.864	22.41	1.09	7.00	0.080	1.14
CT	NA	Shelf	39.844	-70.737	14.5	0.55	0.61	0.002	0.39
ME	Gulf of Maine	Shelf	42.845	-70.478	20.3	1.23	0.65	0.001	0.22

DOS: dissolved organic sulfur; diss. HgT; dissolved total Hg; diss. MeHg: dissolved MeHg; %MeHg: percent MeHg of HgT

Table S2. Comparison of MeHg stability constants with specific low molecular mass (LMM) thiol containing compounds, non-thiol containing compounds, and dissolved organic matter (DOM).

log K	Ligand Source	Experimental Ligand	Reference
16.1-17.4	DOM	Coastal DOM	This study
16.76	LMM thiol	N-acetylpenicillamine	Reid & Rabenstein, 1981
16.94	LMM thiol	Penicillamine	
16.67	LMM thiol	Cysteine	
16.12	LMM thiol	Mercaptoacetic Acid	
5.22	Cl-	Chloride	Loux, 2015
7.57	Amine	Methylamine	Skyllberg et al., 2003
10.39, 14.84	DOM	SRFA	Amirbahman et al., 2002
13.20-14.54	DOM, Strong sites	River/Lake DOM	Hintelmann et al., 1997
12.15-13.07	DOM, Weak sites	River/Lake DOM	
16.3-16.7	DOM	Soil DOM	Qian et al., 2002
16.0-16.7	DOM	Soil DOM	Karlsson & Skyllberg, 2003

SRFA: Suwanee River fulvic acid

Table S3. Chemical reactions and stability constants used in the WinSGW speciation software.

Equation	Reaction	Log *K*	Reference
(2)	$MeHg^+ + DOM\text{-}RS^- = MeHg(DOM\text{-}RS)$	determined	This study
(3)	$MeHg^+ + Nacpen^- = MeHg(Nacpen)$	16.76	Reid & Rabenstein, 1981
(4)	$Nacpen\text{-}H = Nacpen^- + H^+$	-9.6	Liem-Nguyen et al., 2017
(5)	$DOM\text{-}RSH = DOM\text{-}RS^- + H^+$	-9.6	See text
(7)	$MeHg^+ + Cl^- = MeHgCl$	5.4	Loux, 2015
(8)	$MeHg^+ + H_2PO_4^- = MeHgHPO_4^- + H^+$	-1.7	Ingman & Liem, 1974
(9)	$MeHg^+ + H_2O = MeHgOH + H^+$	-4.5	Loux, 2015
(10)	$MeHg^+ + cell\text{-}RS^- = MeHg(cell\text{-}RS)$	16.67	Reid & Rabenstein, 1981
(11)	$cell\text{-}RSH = cell\text{-}RS^- + H^+$	-9.6	See text
(12)	$H_2O = OH^- + H^+$	-13.7	

Table S4. Calculated chemical speciation of MeHg (expressed as % of total MeHg concentration) for each species at each study site, and the Cl^- concentration and pH used in the modeling (SI Text Model 1, Table S3).

	Site	Cl^- (mM)	pH	MeHgOH (%)	MeHgCl (%)	MeHg(DOM-RS) (%)
Marsh	SR	62	7.6	0.01	0.06	99.92
	BC	73	7.6	0.00	0.01	99.99
	BI	437	8.1	0.00	0.03	99.97
	SI	520	8.2	0.00	0.03	99.96
River	OS	9.4	7.5	0.03	0.03	99.92
	PAW	7.8	7.5	0.02	0.01	99.97
	HC	34	7.5	0.03	0.09	99.88
	OR	73	7.6	0.03	0.15	99.84
Estuary	CM	500	8.2	0.03	0.26	99.70
	NB	339	8.0	0.01	0.10	99.89
	CI	436	8.1	0.02	0.19	99.79
	ST	276	7.9	0.00	0.04	99.96
	CTR	325	7.9	0.06	0.58	99.36
	NBD	503	8.2			
	PI	462	8.2			
	CP	151	7.7	0.00	0.04	99.95
	MP	504	8.2			
	M	229	7.8	0.08	0.82	99.09
Shelf	SB	562	8.2	0.07	0.61	99.32
	GOM	495	8.2	0.04	0.32	99.63

Table S5. Relative concentrations (% of total S) of eight pseudo-components of S species deconvoluted from S XANES spectra by Gaussian distributions. Energies for each component are given in Figure S9. The total organic reduced sulfur pool is represented by the sum of RSSR, RSR and RSH functionalities, where R denotes an organic C backbone structure.

	% of Total Sulfur																			
**Site Name**	**BC**	**BI**	**CI**	**CM**	**CP**	**CTR**	**GOM**	**HC**	**LIS**	**M**	**MP**	**NB**	**NBD**	**OR**	**OS**	**PAW**	**PI**	**SI**	**SR**	**ST**
FeS	0	0	0	0	0	0	0	0	0	0	0	0	0	0	0	0	0	0	0	0
FeS2+S0	0	7.7	0	0	0	0	0	0	0	0	0	0	0	0	0	0	0	7.5	0	0
RSSR	21.2	28.2	20.8	21.4	19.3	21.9	13.7	20.1	12.9	18.7	13.0	14.7	21.3	20.5	20.6	22.0	23.6	22.2	22.2	22.5
RSH+RSR	24.6	25.9	22.8	21.4	22.5	25.0	18.1	28.0	15.0	27.6	20.6	22.4	21.9	27.6	27.7	29.0	22.2	29.6	22.2	23.9
Sulfoxide	4.5	2.8	5.0	2.8	4.4	3.9	3.3	4.4	3.1	3.9	3.7	4.3	3.8	5.0	5.5	4.4	4.1	3.6	5.6	4.4
Sulfone	9.9	3.7	5.6	4.8	8.0	6.0	6.0	7.2	7.5	6.0	5.3	9.3	6.3	6.9	8.0	6.4	5.6	4.4	8.1	8.0
Sulfonate	26.5	19.8	29.5	30.7	31.5	27.8	37.8	27.5	38.4	29.5	35.3	31.4	29.4	27.6	26.4	26.5	29.2	21.6	29.0	27.1
Sulfate	13.3	11.9	16.2	18.8	14.2	15.4	21.2	12.8	23.0	14.2	22.2	17.9	17.2	12.4	11.9	11.6	15.4	11.0	13.0	14.1

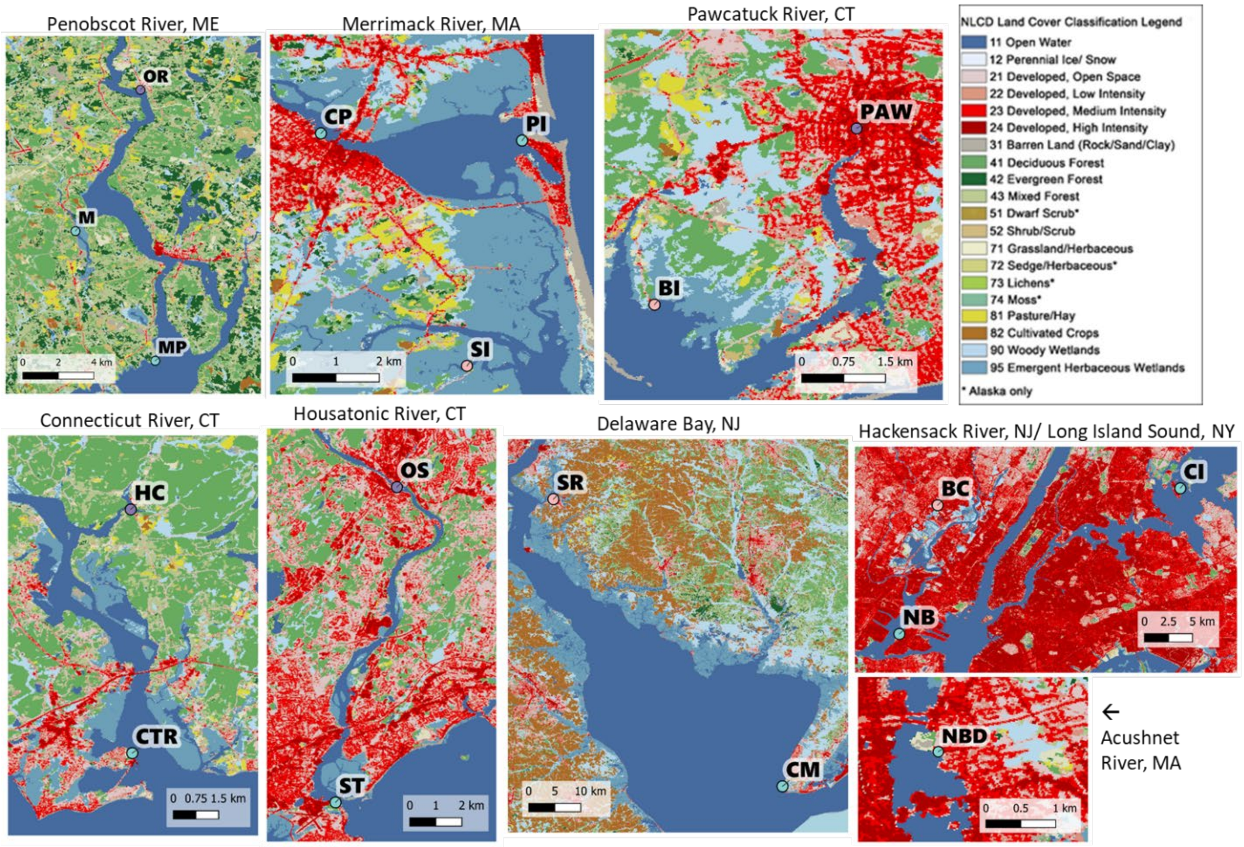

Figure S1. Land use maps from each onshore sampling site. Sites are colored by type of system: Marsh, pink; River, purple; Estuary, blue; Shelf not shown (see further Tables 1 and S1 and Figure 1). Maps were created using QGIS. Land use was downloaded from the MRLC national land cover database 2016 [9], and layered over the Stamen Terrain Background map tiles by Stamen Design, under CC BY 4.0. Data by OpenStreetMap, under ODbL. Source data are provided as a Source Data file.

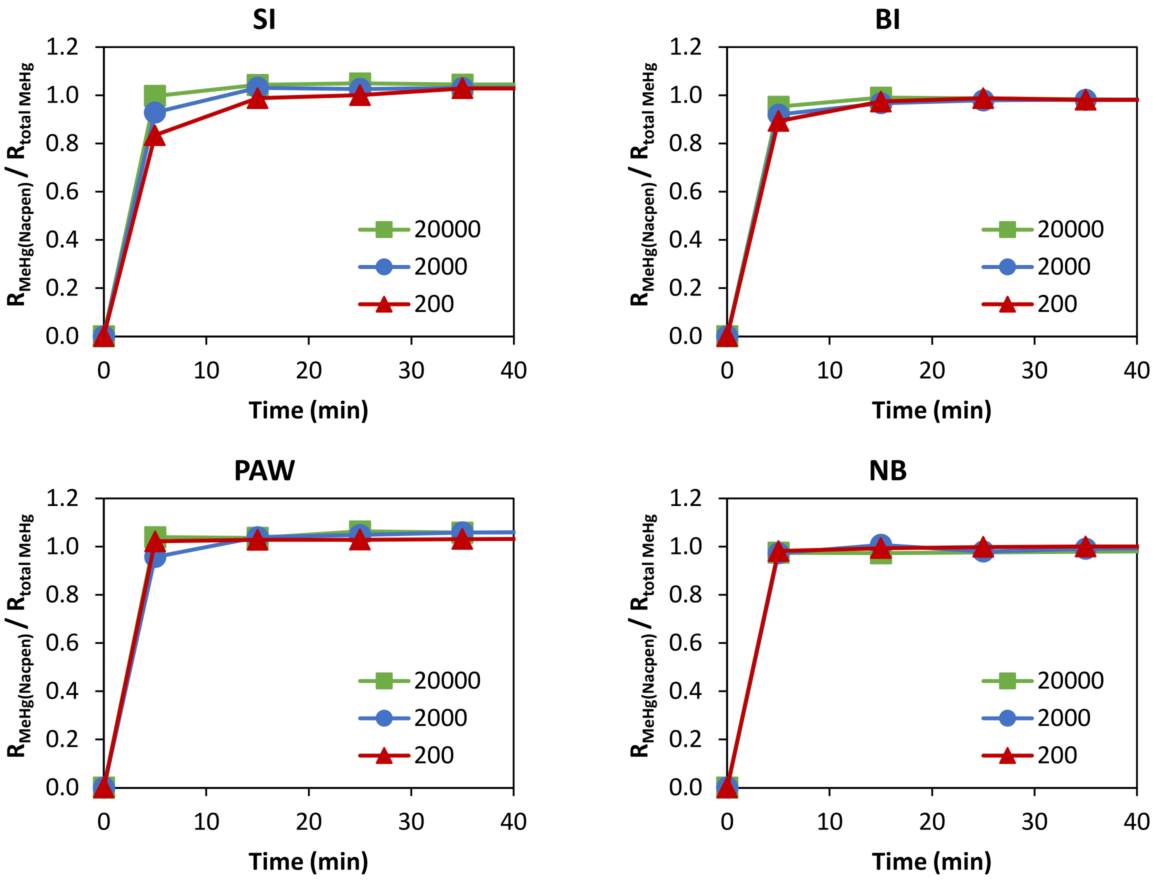

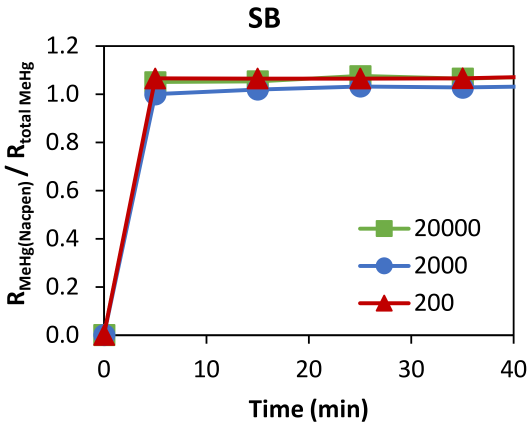

	k_f (min^{-1})	k_r (min^{-1})	turnover (min)
**SI**	0.30	0.04	3.0
**BI**	0.39	0.06	2.2
**PAW**	>0.50	0.04	<1.8
**NB**	>0.61	0.04	<1.5
**SB**	>0.65	0.04	<1.5

Figure S2. MeHg ligand exchange kinetics. The $^{204}Hg/^{200}Hg$ isotope ratio of the MeHg(Nacpen) complex ($R_{MeHg(Nacpen)}$) divided by the $^{204}Hg/^{200}Hg$ isotope ratio of total MeHg ($R_{total\ MeHg}$) over time measured in the kinetic experiment for marsh (SI and BI), river (PAW), estuarine (NB) and shelf (SB) sites. The individual lines represent the different concentrations of competing ligand (20000, 2000 or 200 nM Nacpen) tested in the experiment. At equilibrium $R_{MeHg(Nacpen)}$ / $R_{total\ MeHg}$ = 1.0 and a deviation from 1.0 at steady-state conditions indicates the presence of "non-exchangable" MeHg pools. The Inserted data table shows calculated rate constants for the forward (k_f) and backward (k_r) ligand exchange reactions using equation (6), and the turnover calculated as: turnover = $(k_f + k_r)^{-1}$. Source data are provided as a Source Data file.

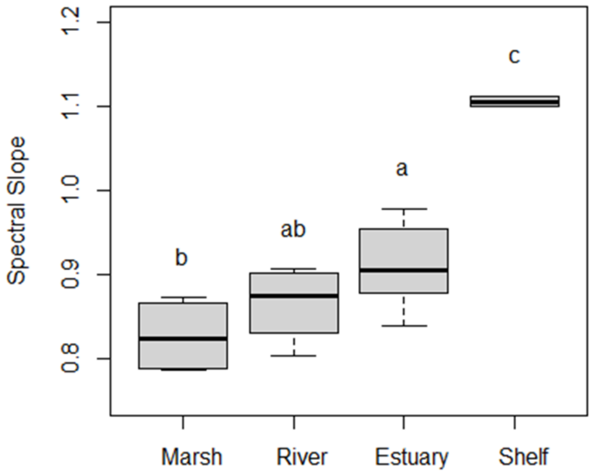

Figure S3. Spectral slope ratios at each site plotted by site grouping. A higher spectral slope (i.e. the ratio of absorption spectra in the spectral slope regions 275-295 nm: 350-400 nm) indicates a smaller molecular weight dissolved organic matter. Significant differences between the site groupings are noted by the letters above the boxes ($p< 0.05$). The top and bottom edge of each box correspond to the first and third data quartiles, respectively, while the center line represents the data mean. Whiskers extend to the furthest data points. The individual site data can be found in the Source Data file.

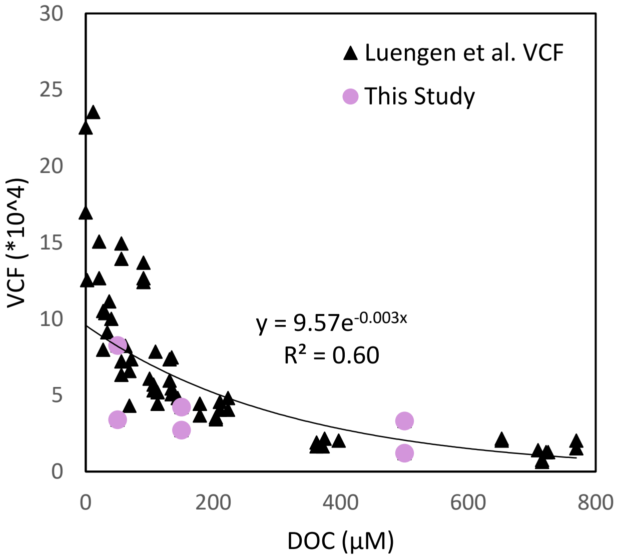

Figure S4. MeHg volume concentration factors. Comparison between the volume concentrations factors measured by Luengen et al. [10] and those determined in this study. The model fit includes both studies. Source data are provided as a Source Data file.

Case A

MeHg(DOM-RS) + H^+= $MeHg^+$ + DOM-RSH K_6 (S1a)

$MeHg(cell)^{n+1}$ = $MeHg^+$ + $cell^n$ K_7 (S1b)

$K_8 = K_6/K_7$ (S1c)

MeHg(DOM-RS) + $cell^n$ + H^+ = $MeHg(cell)^{n+1}$ + DOM-RSH K_8 (S1d)

K_8 = $[MeHg(cell)^{n+1}]$[DOM-RSH] / [MeHg(DOM-RS)]$[cell^n][H^+]$ (S1e)

VCF = $[MeHg(cell)^{n+1}]$ / [MeHg(DOM-RS)] = K_8 $[cell^n]$ $[H^+]$ / [DOM-RSH] (S1f)

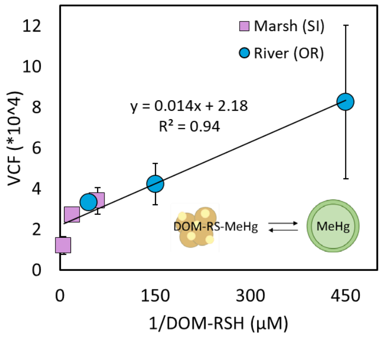

Case B

$U = f_{RS}U_{RS} + (1-f_{RS})U_{Cl}$ (S2a)

$[MeHg(cell)^{n+1}]_t = U \times [MeHg(aq)]_{t=0} \times t$ (S2b)

$VCF = [MeHg(cell)^{n+1}]_t / [MeHg(aq)]'_{t=0}$ (S2c)

Where f_{RS} is the fraction of the MeHg that is MeHg(DOM-RS), U_{RS} is the uptake rate constant of MeHg(DOM-RS), U_{Cl} is the uptake rate constant of MeHgCl, and U is the overall MeHg uptake rate constant (amol μm^{-3} hr^{-1} nM^{-1}). $[MeHg(cell)^{n+1}]_t$ is the MeHg concentration accumulated in cells at time t (amol μm^{-3}), $[MeHg(aq)]_{t=0}$ is the total dissolved MeHg concentration at time 0 (nM), t is the time (h) and $[MeHg(aq)]'_{t=0}$ is the total dissolved MeHg concentration at time 0 (amol μm^{-3}).

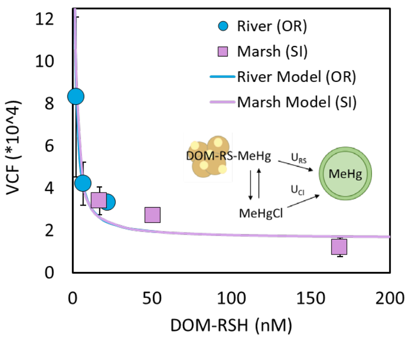

Figure S5. Models for cellular uptake of MeHg by phytoplankton. Case A and Case B represent two methylmercury uptake models tested against the empirical uptake experiment results (the circle and square points in both figures). In case A, the black line and equation represent a linear regression model fit of the uptake experiment results. In case B, the colored lines represent model-generated data based on the uptake experimental conditions. Error bars represent standard deviation (n = 3, except for site SI with lowest DOM-RSH and DOC concentration where n=2) and are smaller than visible in some cases. Source data are provided as a Source Data file.

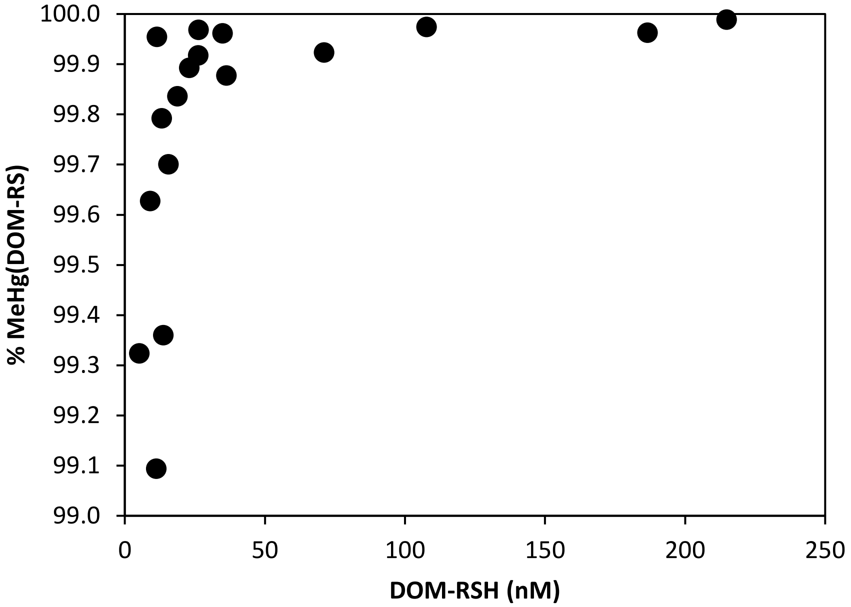

Figure S6. The *in situ* speciation of MeHg at the study sites. Percent fraction of MeHg(DOM-RS) complexes of total MeHg in water versus total DOM-RSH concentration for all study sites (Table S4). Source data are provided as a Source Data file.

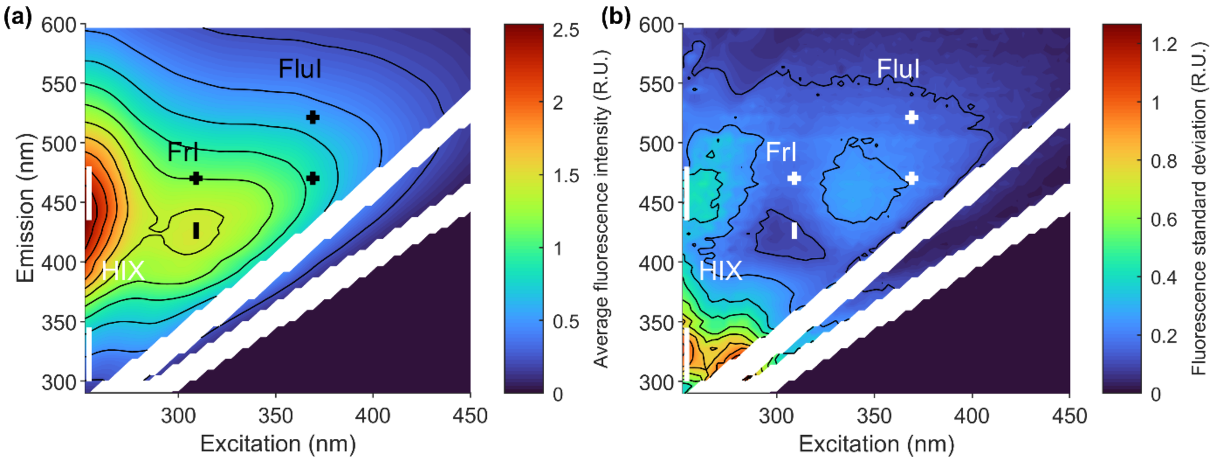

Figure S7. Overview of the raw fluorescence excitation-emission matrices (EEMs) of samples in this study. (a): average fluorescence across all samples. (b): standard deviation of fluorescence across all samples. The fluorescence in each EEM was normalized to its sum prior to the calculation of the standard deviation. Superimposed crosses and lines in the left-upper portion of the figures show the position of the three fluorescence indices used in this study (HIX: humification index (next to y-axis), FrI: freshness index (center), FluI: fluorescence index (right). Source data are provided as a Source Data file.

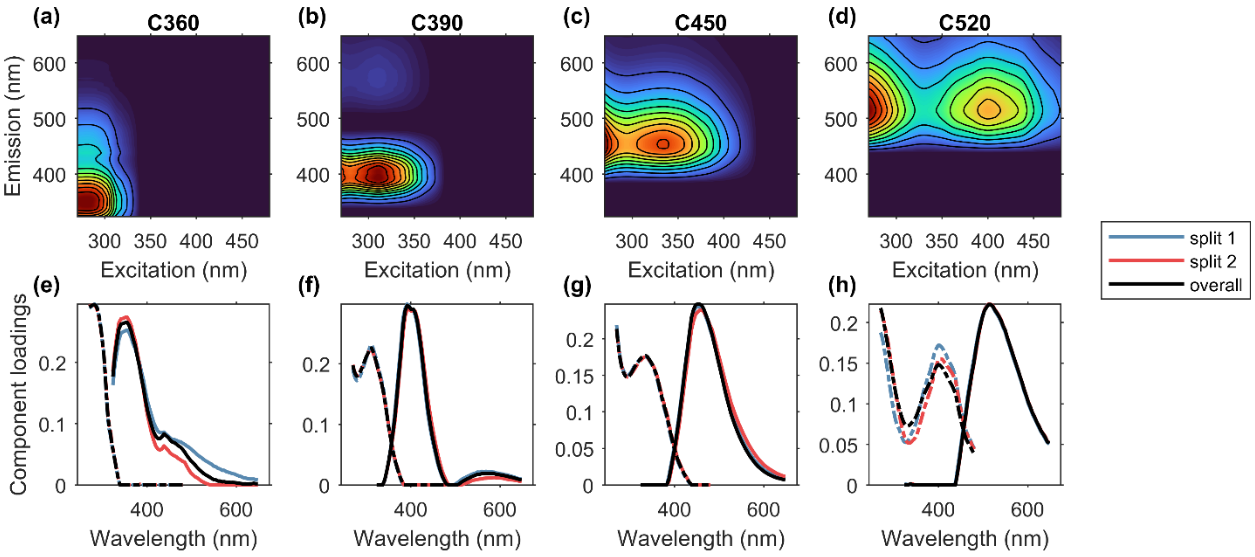

Figure S8. Overview of the four-component Parallel Factor Analysis (PARAFAC) model. (a)-(d): Component loadings as excitation-emission matrix. (e)-(h): Component excitation (dashed lines) and emission (solid lines) loadings as line plots. The results of the splithalf-validation are superimposed in different colors. Source data are provided as a Source Data file.

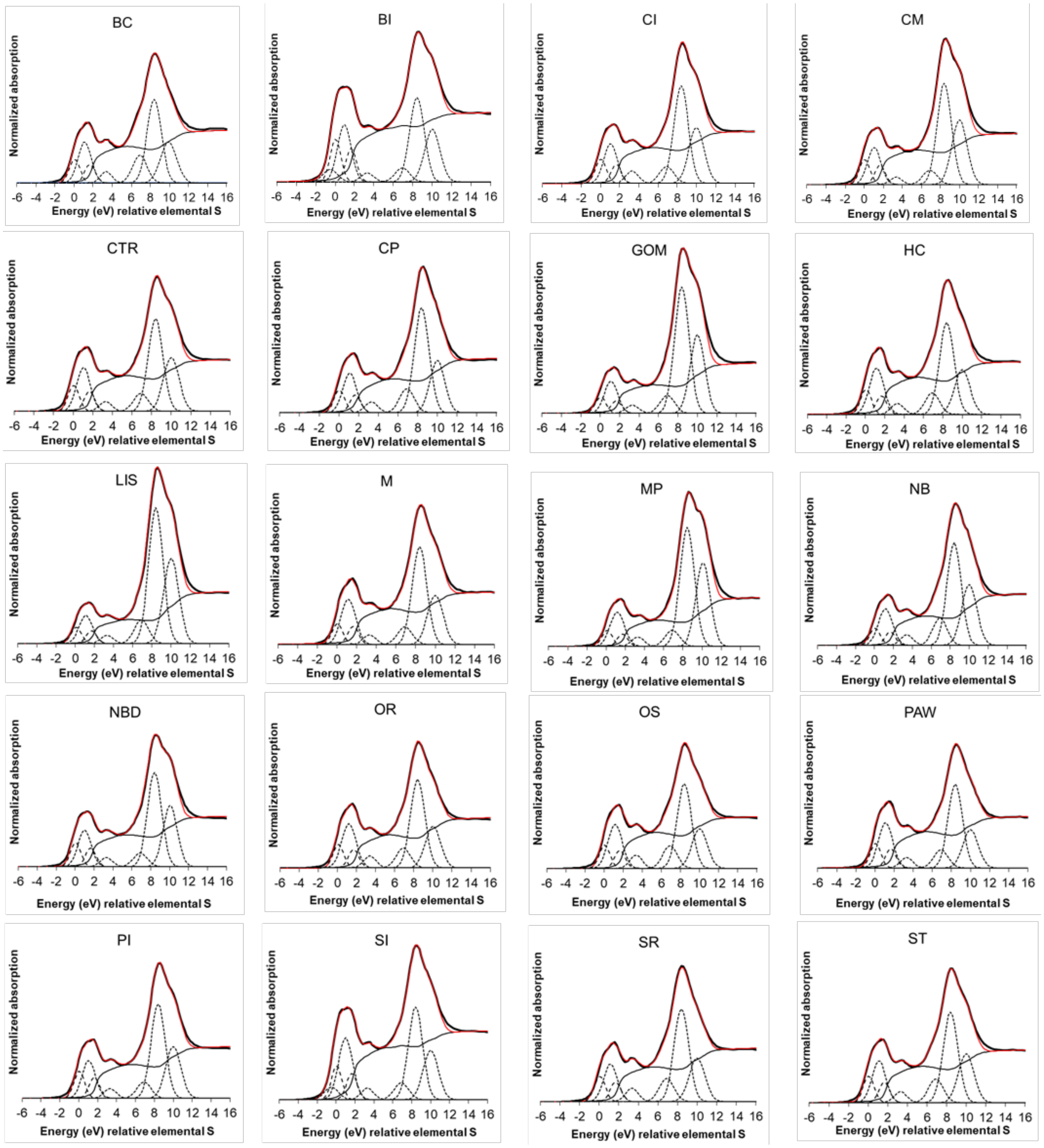

Figure S9. Sulfur K-edge XANES spectra of the extracted DOM. Spectra are deconvoluted into eight pseudo-components (dashed black lines) by use of Gaussian distributions. From left to right: FeS_2+S^0 (one peak centered at -0.5 eV), RSSR (two peaks at 0.05 and 1.65 eV), RSH+RSR (one peak at 1.1 eV), sulfoxide (one peak at 3.3 eV), sulfone (one peak at 6.9 eV), sulfonate (one peak at 8.4 eV) and ester-sulfate (one peak at 10.0 eV). The thin solid black line is background, the thick black line full spectrum and the red line is the full model fit. The method for deconvolution is described in Yekta et al. [11]. Source data are provided as a Source Data file.

SI Text

Chemical Speciation Modeling

The chemical speciation of MeHg in experimental solutions and natural samples was calculated using the WinSGW software [12]. The chemical reactions used are shown in Table S3. Ionic strength was applied using a Debye-Hückel model ($\log f = -Az^2 \sqrt{I}$, $A = 1.82 \times 10^6 (\varepsilon T)^{-3/2}$, T = Temperature, ε= Dielectric constant, I= ionic strength). The temperature was set to 25 degrees C for all models.

Reactions 4, 5 and 11 describe the acid dissociation reaction of the thiol groups in Nacpen, and associated with DOM and cell membranes, respectively. We used the log *K* value of -9.6 determined for Nacpen [13] for all three types of thiols in our models. Although the exact log *K* values for the DOM-RSH and diatom cells used in our study are not known, our approach is reasonable given that reported log *K* for thiols associated with various organic matter typically vary in the range -9 to -10 [14,15] and a log *K* for cell membrane thiols of -9.5 has been reported for bacterial cells [16]. For reaction 11 we used the log *K* value for the MeHg complex with cysteine [1], which is the expected thiol functionality in cell membrane associated proteins.

Model 1 – Determination of log K for MeHg(DOM-RS)

Model 1 was used in the determination of log *K* for MeHg(DOM-RS) for the different DOM samples and the model included reactions 2-9 and 12 in Table S3. The exact total MeHg concentration was determined for each sample by GC-ICPMS and was approximately 100 nM. The exact total DOM-RSH concentration was calculated for each sample based on the added DOM amount and the measured DOM-RSH/OC content and was in the range 0.15 to 2.9 μM. The Nacpen concentration was varied in the concentration range 0.05-20 μM (seven different concentration per DOM sample). Additional input variables were 100 nM Cl^- (from MeHgCl spike), 5 mM $H_2PO_4^-$, and a pH of 7.5.

Model 2 – Chemical speciation of MeHg in natural aquatic systems

Model 2 was used to determine the chemical speciation of MeHg in the natural environment of all the study sites and the model included reactions 2, 5, 7, 9 and 12 in Table S3. The specific log *K* value for reaction 2 determined for the individual DOM samples was used to model MeHg speciation at the corresponding site. The dissolved MeHg concentration was measured at each site (Table S1). The DOM-RSH concentration (nM) at each site was determined by multiplying the measured DOC concentration at

the site with the determined DOM-RSH/DOC content for the corresponding extracted DOM sample (Table 1). The Cl^- concentration (mM) at each site was calculated from the determined salinity (Table 1) as: [Cl^-(mM)] = [salinity(psu)]/(0.00180665 × 35.45) [17]. The pH at each site was estimated by the measured salinity (Table 1) as: pH = 0.02195 × [salinity(psu)] + 7.49. This relationship was made assuming conservative mixing between the lowest salinity sites with a pH of 7.5 and the highest salinity sites with a pH of 8.2. Therefore, the calculated pH varied from 7.5 to 8.2 for the lowest to highest salinity sites.

Model 3 – Chemical speciation of MeHg in cellular uptake experiment

Model 3a was used to describe cellular uptake of MeHg via passive diffusion of MeHgCl without interaction with cell surface ligands. The model included reactions 2, 5, 7, 9 and 12 (i.e. same as model 2). The total MeHg concentration was 10 pM, the DOM-RSH was variable in the ranges 17-170 and 2.2-22 nM for the two sites SI and OR, respectively. The Cl^- concentration was 500 mM and pH was 8.2.

Model 3b was used to describe cellular uptake of via MeHg binding to cell surface thiol groups (cell-RSH) followed by cellular internalization. In addition to the reactions of Model 3a, also reactions 10 and 11 were included. In addition to the concentration input values for Model 3a, a total cell-surface thiol content of 100 nM was estimated using a measured density of ~1 fmol/cell for *T. Pseudonana* [18] a total cell number of 2.4×10^7 cells and an experimental assay volume of 200 ml.

Uptake Equations

Case A

MeHg(DOM-RS) + H^+= $MeHg^+$ + DOM-RSH $\quad K_6$ $\quad$ (S1a)

$MeHg(cell)^{n+1}$ = $MeHg^+$ + $cell^n$ $\quad K_7$ $\quad$ (S1b)

$K_8 = K_6/K_7$ $\quad$ (S1c)

MeHg(DOM-RS) + $cell^n$ + H^+ = $MeHg(cell)^{n+1}$ + DOM-RSH $\quad K_8$ $\quad$ (S1d)

$K_8 = [MeHg(cell)^{n+1}][DOM\text{-}RSH] / [MeHg(DOM\text{-}RS)][cell^n][H^+]$ $\quad$ (S1e)

$VCF = [MeHg(cell)^{n+1}] / [MeHg(DOM\text{-}RS)] = K_8 [cell^n] [H^+] / [DOM\text{-}RSH]$ $\quad$ (S1f)

Case B

$$U = f_{RS}U_{RS} + (1\text{-}f_{RS})U_{Cl} \quad \text{(S2a)}$$

$$[MeHg(cell)^{n+1}]_t = U \times [MeHg(aq)]_{t=0} \times t \quad \text{(S2b)}$$

$$VCF = [MeHg(cell)^{n+1}]_t \,/\, [MeHg(aq)]'_{t=0} \quad \text{(S2c)}$$

Where f_{RS} is the fraction of the MeHg that is MeHg(DOM-RS), U_{RS} is the uptake rate constant of MeHg(DOM-RS), U_{Cl} is the uptake rate constant of MeHgCl, and U is the overall MeHg uptake rate constant (amol μm^{-3} hr^{-1} nM^{-1}). $[MeHg(cell)^{n+1}]_t$ is the MeHg concentration accumulated in cells at time t (amol μm^{-3}), $[MeHg(aq)]_{t=0}$ is the total dissolved MeHg concentration at time 0 (nM), t is the time (h) and $[MeHg(aq)]'_{t=0}$ is the total dissolved MeHg concentration at time 0 (amol μm^{-3}).

Supplementary references

1. Reid, R. S. & Rabenstein, D. L. Nuclear magnetic resonance studies of the solution chemistry of metal complexes . XVII . Formation constants for the complexation of methylmercury by sulfhydryl- containing amino acids and related molecules. *Can. J. Chem.* **59**, 1505–1514 (1981).
2. Loux, N. T. An assessment of thermodynamic reaction constants for simulating aqueous environmental monomethylmercury speciation. *Chem. Speciat. Bioavailab.* **19**, 183–196 (2015).
3. Skyllberg, U., Qian, J., Frech, W., Xia, K. & Bleam, W. Distribution of mercury , methyl mercury and organic sulphur species in soil , soil solution and stream of a boreal forest catchment. *Biogeochemistry* **1**, 53–76 (2003).
4. Amirbahman, A., Reid, A. L., Haines, T. A., Kahl, J. S. & Arnold, C. Association of methylmercury with dissolved humic acids. *Environ. Sci. Technol.* **36**, 690–695 (2002).
5. Hintelmann, H., Welbourn, P. M. & Evans, R. D. Measurement of complexation of methylmercury (II) compounds by freshwater humic substances sing equilibrium dialysis. *Environ. Sci. Technol.* **31**, 489–495 (1997).
6. Qian, J. *et al.* Bonding of methyl mercury to reduced sulfur groups in soil and stream organic matter as determined by x-ray absorption spectroscopy and binding affinity studies. *Geochim. Cosmochim. Acta* **66**, 3873–3885 (2002).
7. Karlsson, T. & Skyllberg, U. Bonding of ppb levels of methyl mercury to reduced sulfur groups in soil organic matter. *Environ. Sci. Technol.* **37**, 4912–4918 (2003).
8. Ingman, F. & Liem, D. H. Solvent extraction studies on the hydrolysis and complex formation of

methylmercury(II) with phosphate ions. *Acta Chem. Scand. A* **28**, 947–956 (1974).

9. Wickham, J., Stehman, S.V., Sorenson, D.G., Gass, L. & Dewitz, J.A. Thematic accuracy assessment of the NLCD 2016 land cover for the conterminous United States. *Remote Sensing of Environment* **257**, 112357 (2021).
10. Luengen, A. C., Fisher, N. S. & Bergamaschi, B. A. Dissolved organic matter reduces algal accumulation of methylmercury. *Environ. Toxicol. Chem.* **31**, 1712–1719 (2012).
11. Yekta, S. S., Gustavsson, J., Svensson, B. H. & Skyllberg, U. Sulfur K-edge XANES and acid volatile sulfide analyses of changes in chemical speciation of S and Fe during sequential extraction of trace metals in anoxic sludge from biogas reactors. *Talanta* **89**, 470–477 (2012).
12. Eriksson, G. An algorithm for the computation of aqueous multicomponent, multiphase equilibria. *Anal. Chim. Acta* **112**, 375–383 (1979).
13. Liem-Nguyen, V., Skyllberg, U., Nam, K. & Björn, E. Thermodynamic stability of mercury (II) complexes formed with environmentally relevant low-molecular-mass thiols studied by competing ligand exchange and density functional theory. *Environ. Chem.* **14**, 243–253 (2017).
14. Skyllberg, U. Competition among thiols and inorganic sulfides and polysulfides for Hg and MeHg in wetland soils and sediments under suboxic conditions: Illumination of controversies and implications for MeHg net production. *J. Geophys. Res.* **113**, 1–14 (2008).
15. Liem-Nguyen, V., Skyllberg, U. & Björn, E. Thermodynamic modeling of the solubility and chemical speciation of mercury and methylmercury driven by organic thiols and micromolar sulfide concentrations in boreal wetland soils. *Environ. Sci. Technol.* **51**, 3678–3686 (2017).
16. Mishra, B. *et al.* Stoichiometry of mercury-thiol complexes on bacterial cell envelopes. *Chem. Geol.* **464**, 137–146 (2017).
17. Kester, D., Duedall, I., Connors, D. & Pytkowicz, R. Preparation of artificial seawater. *Limnol. Oceanogr.* **12**, 176–179 (1976).
18. Fisher, N. S., Boh, M. & Teyssie, J. Accumulation and toxicity of Cd, Zn, Ag, and Hg in four marine phytoplankters. *Mar. Ecol. Ser.* **18**, 201–213 (1984).
